# Hospital and Institutional News

**Published:** 1918-10-12

**Authors:** 


### HOSPITAL AND INSTITUTIONAL NEWS.

### A HAPPY COMPROMISE.

In response to the appeal referred to last week, the authorities of the Hospital Saturday Fund are pleased to announce that a happy compromise was arrived at before the fateful date of the Fund's annual collection on October 12. Mr. Gordon Campbell, Chairman of the Collection Committee of the British Red Cross Society, since wrote:—"I can assure you we should be exceedingly sorry if your well-known Fund suffered by reason of the series of collections in theatres, etc., which the Entertainments Industry Committee is making in our interest throughout the country. We, therefore, have pleasure in agreeing to pay over to you the sum of £1,000 from moneys collected by the Entertainments Industry Committee, thereby equalising the amount your Fund received from theatres, etc., last year. I understand that Mr. Charles Gulliver has offered to give you a *matinée* at the Palladium on October 18, in lieu of the usual 'Hospital Saturday' collection. Whatever amount may be raised by this particular effort will, of course, be a direct contribution to your Fund, and will be over and above the £1,000 which we propose to pass on to you." We are pleased to add that Sir T. Vezey Strong, Chairman, Lord Burnham, Treasurer, and the Collection Committee of this Fund have unanimously decided to accept these two graceful offers with grateful thanks, and to arrange for no collections to be made on behalf of this Fund on "Hospital Saturday," October 12, or during the month of October, at theatres, music-halls, cinemas, etc., with the exception of the *matinée* at the Palladium on October 18. The cheaper tickets may be obtained at the usual booking offices. For ourselves we are happy to think that a solution has been found as simple and generous as it is statesmanlike.

### FRENCH COMMISSIONS FOR AMERICAN WOMEN DOCTORS.

According to a prominent announcement in the *American Red Cross Bulletin*, the first commissions which the French Government has granted to American women doctors have been conferred on Dr. Caroline Finley, Dr. Lee Eward, and Dr. Anna Sholly. They have been made first lieutenants in the French Army, and have further received the *Croix de Guerre* in recognition of their work in a French hospital at the Chateau D'Ognon, near Senlis. In The Hospital of only July 27 we discussed the general question of commissions for medical women and the possibility of the grant of commissioned ranks to women doctors in the English hospitals in France. Qualified women have now had entire charge of military hospitals at home and abroad. The experiment at Endell Street and in France has come to stay. Whether commissions are ultimately granted to women doctors in military hospitals probably depends upon the duration of the war, a phrase in which the most cautious of men is now beginning to take, with due reserve, a degree of active interest.

### A BUSINESS INSPECTOR FOR HOSPITALS.

The ratepayers of New Zealand, as represented by the local authorities, have once more kicked against the ever-increasing levies for hospitals, and the Minister of Public Health (the Hon. G. W. Russell) recently called a meeting of all interested persons thoroughly to exhaust the subject. The Minister expressed the opinion that although the cost of maintaining hospitals had justifiably risen, at the same time the speakers at the conference had convinced him that there was considerable room for improvement in management. He thought that more pressure should be brought to bear upon patients who could but would not pay their fees, and with a view to economy he proposed to appoint a business inspector whose duty it would be to visit every institution in order that practical business management may be introduced into every department of control and every method where money is involved. Where State, municipal support and voluntary effort are mixed together, without system, necessary regulations and adequate administrative control, it cannot be hoped that these ever-recurring squabbles will cease.

### TO WHOM DO DEDUCTIONS FROM WAGES BELONG?

A curious case has arisen in connection with the workmen's contributions to the Pontypool and District Hospital. It raises the question to whom does money which has been stopped from wages for the benefit of a hospital belong before transmission to the institution? Again, has a contributing party the right to make deductions? The matter arose as follows. An accident having occurred at the Eastern Valley Colliery the father of the victim was recommended to take his son to Newport for treatment. He did so, and the expenses of the pair were deducted from the workmen's contributions to the Pontypool Hospital. The committee resolved that it could not accept responsibility for the account, and through its own secretary asked the secretary of the lodge to pay the balance—namely, £2 14s.—to the bank. No reply to this request had been received at the time of the recent meeting, where a noteworthy passage of debate occurred. Why, one speaker asked, was the money not deducted from the contribution to the doctors' fund, instead of from the contribution to the hospital? To this Mr. Arscott replied: "The doctors' money we cannot touch; the hospital money we can." Since the Pontypool Hospital never received the boy as a patient and indeed had no official concern with the case, the men's action is revealed as hasty and unconsidered. The action of the board has been supported by the governors, and if the men are not willing to bear the expense they should take counsel with the person who first recommended the journey and see if no fund exists which could properly contribute to the cost.

### SCOTCH DOCTORS AND THE FRANCO-SERBIAN OFFENSIVE.

The Elsie Inglis Unit is the only field hospital attached to the Jugo-Slav Division, and now that the Franco-Serbian offensive is developing, the Scottish Women are again in the fighting line. The hospital camp was, when the last news came through, at Dragomantsi, north of Vodena, and a month ago a new station, served by motor ambulances, was established still nearer to the Front. Miss Elinor Rendel, M.B. Lond., has gone out to Macedonia as junior surgeon to the Elsie Inglis Unit of the Scottish Women's Hospitals. Miss Rendel has already seen active service. She was a student at the London School of Medicine when she went out to Russia in August, 1916, with Dr. Elsie Inglis. As dresser and *x*-ray assistant, she worked on the Russo-Rumanian front under Dr. Chesney, at the front line dressing stations, and gained much campaigning experience before she returned home a year later to take her degree. To meet the increased drain on its resources caused by the fierce fighting, medical and surgical stores need constant renewal. The treasurers of the London Units are appealing for funds. Lady Cowdray or Miss T. Gosse are open to receive contributions at 66 Victoria Street, S.W. 1.

### BE CAREFUL OF THE OBELISK!

Most towns possessing any public spirit are casting round for some suitable way in which to commemorate the war. We are glad to note that Peterborough decided that its memorial should be a new hospital, which is to cost from £50,000 to £75,000. Down in Dorsetshire, Poole has been hovering between two ideas—an obelisk in the park and a convalescent home. Only powerful advocacy seems to have prevented the latter plan from being overthrown. In the event, the two proposals have been combined, with a marked bias towards the obelisk. Let us pray that the wise men at Poole will remember that, though we have no case against the æsthetic memorial, the question remains whether an obelisk is to be regarded as a worthy example of it. On the other hand, a convalescent home is an example of a utilitarian memorial, and it is easier for the average man to decide whether it fulfils its purpose than, as a member of a committee, to determine the degree of beauty which is necessary for an obelisk before it can be described for certain as a joy for ever to all beholders.

### "TRAVAIL OBLIGE."

The North Lonsdale Hospital has just celebrated the jubilee of its establishment, and the event was marked by the opening of a couple of temporary wards which had been erected in the hospital grounds. The institution is indebted to the workpeople at Messrs. Vickers' for consistent and generous support, which in weekly subscriptions, since the war began, has totalled the handsome sum of £12,780. The working-class subscribers should have already received collectively good value for their money, but there is not everywhere this ready recognition of what hospitals are doing for the wage-earning community. The fetish of representation is apt to override the debt of honour first incurred to the hospital. *Noblesse oblige*, and so does Labour!

### THE SECRETARY'S PEN.

Whether, in consequence of Mr. Leonard Rea's example, or because his cultivation of letter-writing with a purpose was only the first symptom of a general tendency, we do not know, but at all events hospital literature in the pamphlet and the press is steadily improving in interest. Mr. C. R. W. Offen has done good work in educating the district served by the Warneford and Leamington Hospital in the relation of the proposed Ministry of Health to in-patients and out-patients; and now Mr. W. Stevenson, Dr. Basil Rhodes' successor at the North Staffordshire Infirmary, has written an excellent letter to the local newspapers, with extracts from correspondence received by him. These extracts show the goodwill of former patients, who have lately sent gifts in money or in kind. The latter includes a gift of ten shillings which may be described as a gift in kind, because it represents the sale of certain vegetables cultivated by a railwayman on his allotment by the line. That is the way to excite and retain local interest.

### A NEW MIDLAND COTTAGE HOSPITAL.

The committee entrusted with the task of forming a cottage hospital in the Halesowen district, near Birmingham, has agreed to purchase Mount House and its grounds at Mucklow's Hill, and proposes to start with eight beds for emergency cases. Cost and equipment are reckoned at £3,000; an income of £1,000 a year is necessary. Meantime the hospital will be offered to the military authorities for the duration of the war, a phrase which now ceases to suggest eternity.

### AN ACCOUCHEUR INDEED!

The recent death of Dr. William Copley Woodhead, the oldest practitioner in Leeds, removes a man who not only spent sixty-nine years in the study and practice of medicine, but whose boast it was that he had assisted at the delivery of ten thousand infants in his own city, apart from the confinements which he attended elsewhere. It is interesting to recall his first appointment, which appears to have been that of private surgeon to the late Sir George Wombwell, of Balaclava fame. He held it for three years before beginning his long career of private practice.

### STONEHENGE AND WILLIAM MORRIS.

The latest thing to turn to the benefit of the work of the Red Cross Society is Stonehenge, the Tara, one might almost say, of Britain. Mr. C. H. E. Chubb, of Bemerton Lodge, Salisbury, who became its purchaser for £6,600 in 1915 from Sir Cosmo Antrobus, and has lived close by it since his infancy, has now, apparently under the influence of an opinion which he respects, offered it as a gift to the nation. It has been accepted by the First Commissioner of Works, Sir Alfred Mond, on behalf of the nation, who is taking the necessary steps to transfer the property to the care of the Ancient

Monuments Board, which, the First Commissioner is sure, "will guard this priceless possession with sedulous care." As a matter of fact, Stonehenge has a market value, though, like other great works of art, it is without price, and—what, commercially speaking, is more—it brings in a revenue. It is this revenue which, on Mr. Chubb's request, the Treasury has agreed to hand over to the British Red Cross Society. No one will grudge the Society this new gift, but some may think that the Society for the Protection of Ancient Buildings might no less properly have been the recipient of the revenue, could this have been arranged. The Society, in its quiet, unobtrusive way, has done valuable work in rescuing ancient buildings from the risks of time and the worse ravages of the restorer. Associated with William Morris from its foundation, and still known as the "Anti-Scrape" by its friends, it has earned the immense prestige which an overt association with Stonehenge would have given to it. But though the eternal fitness of things would have been satisfied by such an association, this fitness is seldom consulted in an age of progress.

### WHERE MR. HOOLEY WAS.

On Wednesday, October 9, the Queen and Princess Mary arranged to visit informally the Papworth Hall Tuberculosis Colony in Cambridgeshire, where fifty discharged soldiers are now accommodated in what was once Mr. Hooley's house. Advanced cases are housed indoors, incipient cases in open-air shelters. Seven trades are being taught, and maimed patients are accommodated in cottages on the estate. The cost of the scheme is £35,000, of which £7,000 has been raised. Its range and system are such as to have attracted the Queen's notice, and we hope that one result of her visit will prove to be the final liquidation of the debt.

### THE "WAR-TIME LUXURY" OF VISITING PATIENTS.

The practice of visiting patients from Southwark who are boarded out in the lunatic asylums and other special institutions has been discussed by the Southwark Board of Guardians. Visiting was opposed on the ground of expense, and as a war-time luxury. Hitherto these visits have been of immense service, inasmuch as they have kept the custodians of the poor in complete touch with those for whom they are primarily responsible, for it had been found that these poor people when sent away were often forgotten, in some cases for years, by their relatives. The visits are a breath from the outside world, which helps to bring together the bereaved and those who should care for them. For many years the Lambeth Guardians did not visit these institutions, and it was only after extreme pressure from the Local Government Board that they appointed committees to visit. The visitations well paid them, for they were able to get in touch with many relatives of the patients, who were not contributing anything towards their support, though well able to do so. On humane and economic grounds alike the practice of visiting should be very carefully considered before it is abolished at the parrot cries of "luxury" and "war." If the majority at Lambeth degenerate into opponents of visiting committees the Guardians should be abolished with all possible speed.

### THE ABUSE OF SANATORIA BY THE MILITARY.

The military mind is prone to let things drift till they are past mending and then to shunt the responsibilities for dealing with a hopeless position on to somebody else. It is a costly and lazy habit, but so long as the cost of delay and the fruits of laziness can be passed on, the military mind is hardly likely to be brought to its senses. A case in point is that of the tuberculous soldier. Many early cases of the disease have been thoughtlessly passed into the Army; many more borderline cases succumbed under the strain of military life and training, but the sick soldier is officially a nuisance, and such a thing as an organised system of sanatorium treatment for early or suspected cases is no part of the Army medical machine. The consequence is that many cases remain for long untreated, perhaps even undiagnosed, and after the waste of precious weeks or months are at last recognised as hopeless, from the military point of view, and therefore to be got rid of like a hot potato. In answer to the question "Where?" the hasty answer comes "To the nearest sanatorium," which, the military mind knows, cannot refuse to admit the most advanced case without incurring odium, though any blame deserves to fall on those who allowed the disease to develop so far without effective treatment. In the present state of our knowledge sanatoria can hope permanently to arrest the disease in early cases only. To bring an advanced case to the doors of an institution which receives patients only in the early stage is to force a choice between a rejection which looks inhuman and an admission harmful to the other patients. For the sight of one (as they suppose) in the like case with themselves who dies after a brief stay in an institution undermines the reputation of the institution, which is the patient's chief hope, and often causes rebellion against the regulations of the treatment, a fatally hasty departure, or the despair which is one of the doctor's worst enemies in the treatment of pulmonary tuberculosis. The advanced case needs its own class of institution, and the advanced military case should very rarely occur at all. When it does, the military authorities should be held responsible, and be prevented from "dumping" the unfortunate patient on civil institutions totally unfit for cases in this stage of the disease. Among the sanatoria which have been victimised is Winsley. Probably united action by the sanatoria of the country can alone reduce the practice to reasonable limits.

### STAFFS AND THE CERTIFIED OCCUPATION LIST.

In the new list of certified occupations issued by the Ministry of National Service, for the first time the staffs of hospitals, infirmaries, and similar public institutions are included in the section devoted to public authorities and public Services. Employees of these ages are not liable for medical examination, and should they receive a notice calling upon them to be medically examined they should return it at once to the National Service Office who sent it out, with a written notification from the secretary or a governor of the institution stating their occupation and that they have been similarly employed since January 1. The men who are in the certified list should have been born in or before the year stated. The reservations are as follows:—

| | Grade 1 | Grade 2 |
|---|---|---|
| 572 (*a*) Steward ... ... ... ... ... | 1878 | 1883 |
| 573 (*b*) Pharmacist, dispenser, male nurse, theatre attendant, mortuary attendant, outdoor porter ... ... ... ... | 1880 | 1885 |
| 574 (*c*) All other classes of employees ... | 1875 | 1880 |

In the case of employees who have already been medically examined and placed in either Grade 1 or 2, it will be necessary, if it is desired to retain their services, to lodge appeals at the local tribunals, who should grant them conditional exemption as being in the Certified Occupation List.

### THE CERTIFIED LIST AND MENTAL INSTITUTIONS.

With regard to institutions for lunatics and institutions and homes for mental defectives under the Mental Deficiency Act, 1913, the Certified Occupation List states that a man employed in or about such an institution is to be treated as in a certified occupation if recommended by the Board of Control, but it is laid down that the Board of Control will not, without the concurrence of the Ministry of National Service, issue a certificate of recommendation to any attendant in Grade 1 who was born after 1883, or in Grade 2 who was born after 1888, or to any other employee in Grade 1 who was born after 1878 or in Grade 2 who was born after 1888. It might be pointed out with reference to Grade 3 men of the new military age that there is no necessity to lodge appeals for them for exemption until they have received notice calling them to the Colours, when appeals can be lodged for their retention within seven days of the receipt of the calling-up notice.

### A CRUEL HOAX.

With the best intentions in the world people sometimes do a lot of harm. Here is Mr. J. Hartley Manners, whose "Peg o' My Heart" made his name happily familiar to all of us, writing from New York to the British press a circular-letter in which he makes, at second hand, monstrously ridiculous accusations against English chemists. If there were a grain of truth in his assertions England would certainly not be a safe place either for American or English soldiers; but fortunately there is not a grain of truth in them. "I have received," he says, "first-hand evidence that morphine can be bought freely in certain chemists' shops in England in any quantity without a physician's prescription, and with no obligation on the part of the purchaser of signing 'a poison book'; . . . that as many as 200 persons have been seen waiting in line in front of a chemist's shop to purchase morphine—practically all of them ex-soldiers." The fact of the matter is, to use an expression current in military circles, somebody has been "pulling the leg" of the famous author. It probably came about in this wise: In the days before coupons began there was seen by a confidant of Mr. Manners a long queue of people lined up at a margarine shop; this shop was next door to a chemist's shop, and the friend of Mr. Manners, who was passing on the top of a 'bus, taking a too hasty glance, thought the people were lined up at the chemist's. "What is the 'beatific' gentleman inside doling out to these men?" asked the 'bus passenger who had been reading De Quincey. "Why, morphine, of course." And so he told the whole shameful story to Mr. Manners. To those who know how difficult it is, even sometimes for a doctor, to buy morphine in this country, the whole business is, of course, ludicrous. But it has its serious side, because there are so many people who do not know the facts and may be disposed to believe the story. We are glad to learn from the secretary of the Pharmaceutical Society that he has taken the matter up, and has asked Mr. Manners for further details of the amazing tale.

### THIS WEEK'S DRUG MARKET.

Business has not been particularly active during the week, and it need hardly be said that, in view of recent events, buyers are exercising particular caution. The advice tendered to hospital buyers of drugs and chemicals in last week's report that they should watch the markets very closely can now be emphasised. At the time of writing the effect of the latest developments in the military situation are not very apparent. Quinine prices are still firmly maintained, but when the expected supplies arrive it is not improbable that the value will recede somewhat. Mainly owing to increased cost of labour, manufacturers of bismuth preparations have advanced their prices. The advance is not very substantial, however, as things go nowadays. Citric acid still tends upwards in price, and citrates are quoted at higher rates. As was expected, English refiners of camphor have advanced their quotations, but they are still materially below the prices of Japanese refined. Very high prices are being paid for cascara sagrada, and comparatively little is available. Saffron continues to tend upwards in price. Aspirin is again dearer, phenacetin has ceased its downward tendency, and antifebrin tends upwards in price. Gum ammoniacum is scarcer than ever. Buchu leaves seem likely to be dearer. Further business has been done in menthol and Japanese peppermint oil at the advanced rates. An advance in the value of eucalyptus oil seems probable.

### TO OUR READERS.

**Contributions are specially invited from any of our readers to these columns. They should deal with topical subjects and news. They must be authenticated for the information of the Editor only. The minimum payment if published is 5s. There is no hard-and-fast rule as to space, but notes of about twenty lines in length are preferred.**

